# Prenatal Diagnosis of Small Supernumerary Marker Chromosome 10 by Array-Based Comparative Genomic Hybridization and Microdissected Chromosome Sequencing

**DOI:** 10.3390/biomedicines9081030

**Published:** 2021-08-17

**Authors:** Igor N. Lebedev, Tatyana V. Karamysheva, Eugeny A. Elisaphenko, Alexey I. Makunin, Daria I. Zhigalina, Maria E. Lopatkina, Gleb V. Drozdov, Aleksander D. Cheremnykh, Natalia B. Torkhova, Gulnara N. Seitova, Stanislav A. Vasilyev, Anna A. Kashevarova, Ludmila P. Nazarenko, Nikolay B. Rubtsov

**Affiliations:** 1 Tomsk National Research Medical Center, Research Institute of Medical Genetics, 634050 Tomsk, Russia; darya.zhigalina@medgenetics.ru (D.I.Z.); maria.lopatkina@medgenetics.ru (M.E.L.); drozdovglebv@mail.ru (G.V.D.); a.cheremnykh@medgenetics.ru (A.D.C.); genetics@tnimc.ru (N.B.T.); gulnara.seitova@medgenetics.ru (G.N.S.); stanislav.vasilyev@medgenetics.ru (S.A.V.); anna.kashevarova@medgenetics.ru (A.A.K.); ludmila.nazarenko@medgenetics.ru (L.P.N.); 2 Institute of Cytology and Genetics of the Siberian Branch of the Russian Academy of Sciences, 630090 Novosibirsk, Russia; kary@bionet.nsc.ru (T.V.K.); kanopus@ngs.ru (E.A.E.); rubt@bionet.nsc.ru (N.B.R.); 3 Wellcome Sanger Institute, Cambridge CB101SA, UK; alex.makunin@gmail.com; 4 Department of Cytology and Genetics, Novosibirsk State University, 630090 Novosibirsk, Russia

**Keywords:** array-based comparative genomic hybridization, chromosomal microdissection, single-copy chromosome sequencing, small supernumerary marker chromosome, ring chromosome, prenatal diagnosis, mosaicism

## Abstract

Interpreting the clinical significance of small supernumerary marker chromosomes (sSMCs) in prenatal diagnosis is still an urgent problem in genetic counselling regarding the fate of a pregnancy. We present a case of prenatal diagnosis of mosaic sSMC(10) in a foetus with a normal phenotype. Comprehensive cytogenomic analyses by array-based comparative genomic hybridization (aCGH), sSMC microdissection with next-generation sequencing (NGS) of microdissected library, fluorescence in situ hybridization (FISH) with locus-specific and telomere-specific DNA probes and quantitative real-time PCR revealed that sSMC(10) had a ring structure and was derived from the pericentromeric region of chromosome 10 with involvement of the 10p11.21-p11.1 and 10q11.21-q11.23 at 1.243 Mb and 7.173 Mb in size, respectively. We observed a difference in the length of sSMC(10) between NGS data of the DNA library derived from a single copy of sSMC(10), and aCGH results that may indicate instability and structural mosaicism for ring chromosomes in foetal cells. The presence of a 9 Mb euchromatin region in the analysed sSMC(10) did not lead to clinical manifestations, and a healthy girl was born at term. We suggest that the ring structure of sSMCs could influence sSMC manifestations and should be taken into account in genetic counselling during prenatal diagnosis.

## 1. Introduction

Small supernumerary marker chromosomes (sSMCs) are small additional structurally abnormal chromosomes in karyotypes of some patients. They cannot be identified or characterized unambiguously by conventional banding cytogenetics alone and are generally equal in size to or smaller than chromosome 20 within the same metaphase spread [1]. Some sSMCs have no clinical significance, but others are pathogenic [2]. sSMCs usually consist of chromosomal material derived from the pericentromeric region of one chromosome, but an additional region from the more distal part of the chromosome may also be present [3]. The location of the breakpoints determines the DNA content of sSMCs and their potential pathogenesis, but the relationships between the size of sSMCs and their clinical manifestations are still controversial [4,5]. Furthermore, in the case of breakpoints in both arms of two-armed non-acrocentric chromosomes, the sSMC terminal ends are protected by additional changes, namely, clusters of telomeric repeats or the formation of a ring chromosome. Notably, sometimes sSMCs are present only in some cells, i.e., carriers of sSMCs can be mosaics. The identification and description of mosaicism is a challenge for prenatal and postnatal diagnosis since detecting sSMCs in most tissues requires a large number of biopsies, which is not possible during conventional diagnostics. Additionally, sSMCs can contain regions in which the transcriptional activity of genes is epigenetically regulated. Most likely, the position effect or changes in topologically associated domains (TADs) can influence the clinical manifestation of sSMCs. For sSMCs of 3–5 Mb in size, the phenotype prognosis is usually favourable. However, for longer sSMCs, the dilemma for clinical interpretation and prognosis during prenatal diagnosis is much more complex. Several critical points should be checked in genetic counselling during pregnancy, including the following:

1. The chromosome-derived origin of the sSMC;
2. The DNA content of the sSMC, including its euchromatin and heterochromatin regions;
3. The complete or mosaic form of the sSMC, including all other possible derived karyotypes as a consequence of chromosomal instability;
4. The de novo or inherited nature of the SMC;
5. The structural organization of the sSMC, which may affect transcription-related processes through epigenetic chromatin modifications, TAD reorganization, and particularity of the sSMC territory in the interphase nucleus.

In this study, we report the results of comprehensive cytogenomic analysis of the sSMC(10) observed during prenatal diagnosis in a foetus without detected developmental defects. The obtained results allowed precise determination of the origin, structure, size and ring form of this sSMC derived from the proximal pericentromeric region of chromosome 10 near a 9 Mb euchromatin region. The possible role of the ring structure and instability in the clinical manifestation of the analysed sSMC and its significance for genetic counselling in prenatal diagnosis are discussed.

## 2. Materials and Methods

### 2.1. Case Report

A 34-year-old pregnant woman was referred to genetic counselling for a prognosis of foetal health. It was her second pregnancy from a non-consanguineous marriage without a known history of hereditary diseases in the pedigree. Parental karyotypes were normal according to GTG banding of metaphase chromosomes in both parents. During biochemical testing at 11 weeks of gestation, a slightly elevated level of ß human chorionic gonadotropin (ß-HCG) (148.9 ME/L, 3.537 MoM) and a normal level of pregnancy-associated plasma protein-A (PAPP-A) (7.242 ME/L, 1.591 MoM) were noted. The crown-rump length was 64 mm. The nuchal translucency was 1.50 mm. The corrected individual risks were calculated for trisomy 21 (1:4936), trisomy 18 (1:17,167) and trisomy 13 (<1:20,000). At 20.4 weeks of gestation, hypoplasia of the nasal bone was noted by ultrasonography as a single marker of chromosomal abnormalities, and invasive prenatal diagnosis was recommended. Cordocentesis and G-banding metaphase analysis were performed at 20.5 weeks of pregnancy, and the mosaic karyotype was revealed: mos 47,XX,+mar [18]/46,XX [5]. Ultrasonography at the age of 23.3 weeks confirmed the presence of nasal bone hypoplasia and revealed normal foetal measurements corresponding to gestational age. The head circumference was 213 mm, biparietal diameter—57 mm, fronto-occipital diameter—74 mm, right and left femur length—41 mm, right and left humeral bone length—36 mm, and abdomen circumference—193 mm. Repeated cordocentesis was recommended and performed at 23 weeks of pregnancy for additional comprehensive cytogenomic investigations. Informed consent was obtained. A healthy girl was born at term. The patient’s birth weight was 3530 g (50th centile), her birth length was 54 cm (75–90th centile), her head circumference was 36 cm (75th centile), and her chest circumference was 34 cm (50–75th centile). Her Apgar scores were eight and nine after 1 and 5 min, respectively.

### 2.2. Cytogenetic and Molecular Cytogenetic Studies

Metaphase spreads from cord blood and newborn peripheral blood lymphocytes were obtained, and GTG-banded chromosomes were analysed according to standard protocols [6]. Metaphase and prometaphase spreads for fluorescence in situ hybridization (FISH) were prepared according to [7]. Metaphase spreads for chromosomal microdissection were performed as described previously [8,9].

Chromosomal microdissection was performed by an MR micromanipulator (Carl Zeiss, Jena, Germany) on an Axiovert 10 inverted microscope (Carl Zeiss, Jena, Germany) using a glass needle under a 100× objective. A DNA library was obtained from one copy of isolated sSMCs by degenerate oligonucleotide-primed polymerase chain reaction (DOP-PCR) as described previously [8]. To label the microdissected DNA library with Alexa Fluor 488 or TAMRA, an additional 20 cycles of high-temperature DOP-PCR in the presence of Alexa Fluor 488-5-dUTP (Invitrogen, Waltham, MA, USA) or TAMRA-5-dUTP (Roche, Basel, Switzerland) were carried out [8,9]. The specificity of the whole-chromosomal paint probes for the microdissected sSMC (WCPmar) was confirmed by FISH with the proband’s metaphases. Detection of the origin and content of the sSMC was performed using chromosomal in situ suppression hybridization (CISS-hybridization) with WCPmar on metaphase spreads obtained from peripheral blood lymphocytes of a healthy donor by a protocol described previously [10].

A DNA probe for the detection of telomeric (TTAGGG)n repeat clusters was obtained as previously described [11]. Partial chromosome paint probes specific to 10p11.2q22.2, the centromere-specific D10Z1 probe, and the RP11-225E2 and CELF2/BRUNOLS3 (DiGeorge II (10p14), CytoCell, Cambridge, UK) probes were used to characterize the structure of the sSMC. Locus-specific identifiers were synthesized using long-range PCR by a previously reported protocol [12]. Oligonucleotide primers for the unique sequences of genes *ZNF248* (10p11.21) and *MAPK8* (10q11.22) as well as long-range PCR conditions are presented in the Supplementary Materials (Table S1).

Visualization of the euchromatic region of the sSMC was performed by FISH using a TAMRA-labelled DNA probe prepared by amplification of the fragment of the Alu repeat [9,13].

GTG-banded metaphase chromosomes were analysed using an Axio Scope A1 microscope (Carl Zeiss, Jena, Germany) with Ikaros Karyotyping Systems software, V.5.8.14 (MetaSystems, Altlussheim, Germany). FISH and CISS-hybridization results were visualized and registered with an AXIOPlan2 Imaging microscope (Carl Zeiss, Jena, Germany) equipped with fluorescence-specific filters (CHROMA, Wixon, MI, USA) and a CCD camera at the Core Facility for microscopic analysis of biological objects at the Institute of Cytology and Genetics SB RAS (regulation no. 3054). Image analysis was performed using ISIS5 software (MetaSystems GmbH, Altlussheim, Germany).

### 2.3. aCGH

Array-based comparative genomic hybridization (aCGH) was performed using a SurePrint G3 Human CGH+SNP 4 × 180K Microarray Kit (Agilent Technologies, Santa Clara, CA, USA) with 13 kb overall median probe spacing (11 kb in RefSeq genes) according to the manufacturer’s recommendations. Labelling and hybridization of the patient’s and reference DNA (#5190-3797, Human Reference DNA Female, Agilent Technologies, Santa Clara, CA, USA) were performed using enzymatic labelling and hybridization protocols, v.7.5 (Agilent Technologies, Santa Clara, CA, USA). Array images were acquired with an Agilent SureScan Microarray Scanner (Agilent Technologies, Santa Clara, CA, USA). Data analysis was performed using CytoGenomics Software, v.5.1.2.1 (Agilent Technologies, Santa Clara, CA, USA) and the publicly available Database of Genomic Variants (DGV) resources. Human genome assembly 19 (hg19) and GRCh38 were used to describe the molecular karyotype revealed by aCGH.

aCGH analysis was performed at the Core Facility “Medical Genomics” of the Tomsk National Research Medical Center of the Russian Academy of Sciences using the resources of the biocollection “Biobank of the population of Northern Eurasia”.

### 2.4. Sequencing of the DNA Library from the Microdissected sSMC

The microdissected DNA library of the sSMC prepared by DNA amplification in DOP-PCR was sequenced. The KAPA adapter ligation and library preparation kit (KAPA Single-Indexed Adapter Kit Illumina^®^ Platforms KR1317—v3.17, Saint Louis, MO, USA) was used. The quantity and quality of the library were assessed using an Agilent Bioanalyser, followed by next-generation sequencing (NGS) on an Illumina X Ten platform. The resulting 150 bp paired-end reads were aligned to the human genome (hg38) with HISEQ2 visualized in GENEIOUS (Figure S3). In addition, the data were analysed using the DoppSeq pipeline [14]:

- Adapters and DOP primers were trimmed with cutadapt 2.9 [15];
- Trimmed reads were aligned to the human genome (hg38) with bwa mem 0.7.15 [16]; and
- Mapped reads were filtered (minimum 20 bp hit length, minimum 0 or 20 MAPQ), merged into positions, and used to segment the genome based on distances between positions with DNA copy 1.52.0 [17].

### 2.5. Analysis of Locus Copy Number Variation with Quantitative Real-Time PCR

Target sequences within and outside the chromosomal region homologous to the sSMC according to aCGH and NGS results and specific amplification primers for quantitative real-time PCR assays were selected using Primer 3 software (Table S2) [18]. The partial trisomy of the 10p11.21 and 10q11.21q11.23 euchromatic regions was tested using genomic DNA of the foetal cord blood cells, parental peripheral blood lymphocytes and a control sample using the AriaMX Real-Time PCR System (Agilent Technologies, Santa Clara, CA, USA). Control genomic DNA was obtained from the peripheral blood lymphocytes of a healthy donor. The control gene was *HEXB*, which encodes the beta subunit of hexosaminidase and is located at 5q13 (Table S2).

## 3. Results

### 3.1. sSMC Origin and Content Determined with FISH Techniques

**Figure 1:**
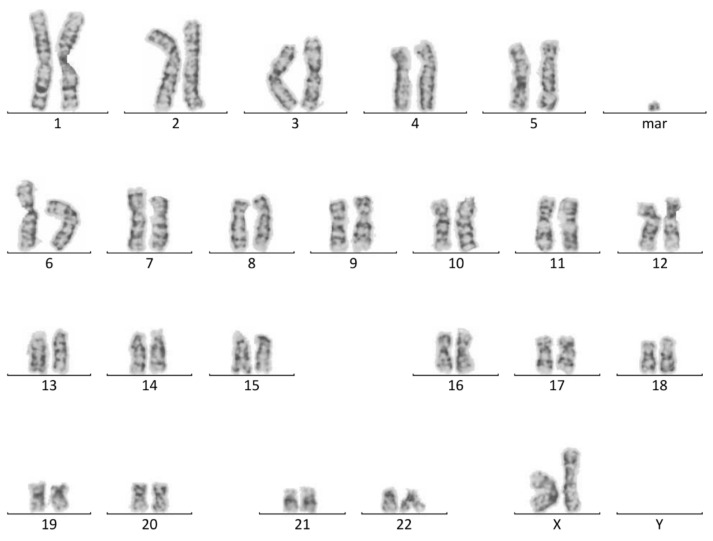
Karyotype 47,ХХ,+mar. GTG banding of metaphase chromosomes from foetal cord blood lymphocytes at 20.5 weeks.

Cytogenetic analysis of GTG-banded metaphase chromosomes from foetal cord blood lymphocytes revealed the sSMC in 78% of metaphase spreads: 47,XX,+mar [18]/46,XX [5] (Figure 1).

**Figure 2:**
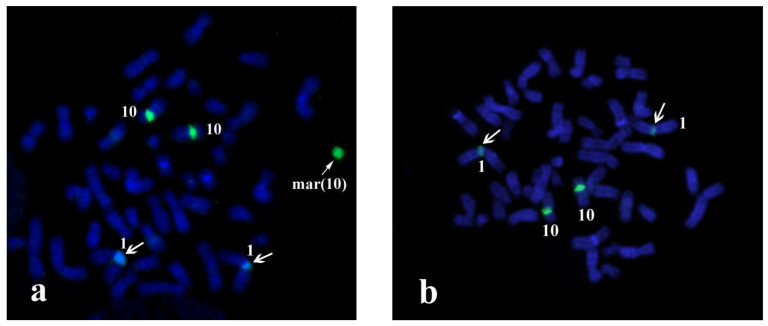
Painting patterns for WCPmar. (**a**) Foetus chromosomes; (**b**) healthy donor chromosomes. Chromosomes 1 (1), 10 (10) and sSMC(mar(10)) are marked; → indicates a signal on the C-region of the q-arm of chromosome 1; → indicates a signal on the sSMC. Chromosomes are stained with DAPI.

Due to the small size of the marker chromosome, a complex strategy for its identification was applied based on independent parallel analysis of the origin and euchromatin content as well as gene’s content of the marker chromosome by microdissection with reverse painting, NGS of the DNA library derived from the sSMC and aCGH with quantitative real-time PCR. Intense WCPmar painting of the sSMC, proximal region of both homologues of chromosome 10, including its pericentromeric region and small proximal euchromatic regions of the short and long arms (Figure 2a), was observed. Less intense WCPmar painting of the C-positive region of the long arm of chromosome 1 (Figure 2a) was observed. The painting patterns of normal chromosomes of the foetus and healthy donor were identical (Figure 2a,b).

In CISS-hybridization, the hybridization of repetitive DNA should be suppressed. However, in our study, DNA probe obtained from microdissected marker chromosome (WCPmar) painting of some regions consisted of DNA repeats, namely, the C-positive region of chromosome 10 and part of the C-positive region of chromosome 1 was observed. An intense signal in the C-positive pericentromeric region of chromosome 10 and its small proximal euchromatic regions allowed us to conclude the origin of the sSMC from the proximal region of chromosome 10. Notably, despite suppression of DNA repeat hybridization, microdissected DNA probes obtained from sSMCs always paint the C-positive pericentromeric region of the original chromosomes. The WCPmar, like the original sSMC, consists mostly of repeated sequences; therefore, the level of suppression of repetitive DNA hybridization during CISS-hybridization appears to be usually insufficient to completely eliminate the signal in the sSMC and in the pericentromeric C-positive region of the original chromosome. Usually, such suppression is sufficient to eliminate the fluorescence signal in the pericentromeric C-positive regions of other chromosomes despite the presence of a certain number of homologous repeats in these regions. In our study, WCPmar painted less intensive the C-positive region of chromosome 1. It probably contains more DNA repeats homologous to DNA of the pericentromeric region of chromosome 10 than C-positive regions of other chromosomes.

In addition to the pericentromeric C-positive region of chromosome 10, the fluorescence signal was detected in the C-negative proximal regions of its p- and q-arms. When analysing the distribution of the fluorescence signal intensity, it is necessary to take into account the phenomenon of chromosome material spreading into neighbouring regions in the process of metaphase chromosome preparation. In the case of CISS-hybridization with WCPmar, the signal in 10q indicated the presence of a C-negative region in the sSMC ranging in size from 5 to 10 Mb derived from the long arm of chromosome 10. However, we cannot exclude that the registered signal provided with WCPmar on 10p is the result of the spreading DNA from the C-positive region in the proximal region of 10p.

**Figure 3:**
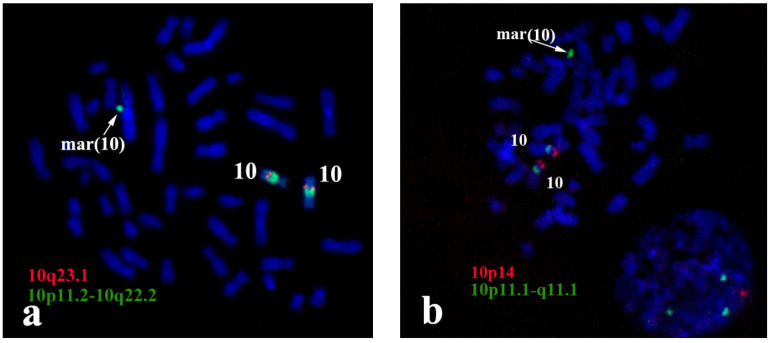
Painting with PCP10q- and FISH with locus-specific DNA probes on foetal metaphase chromosomes. DAPI staining is blue. (**a**) Painting with PCP10q- (green signals) and FISH with BAC clone RP11-225E2 (red signals); (**b**) FISH with D10Z1 (green signals) and CELF2/BRUNOLS3 (red signals) probes.

Additional confirmation of the sSMC origin from the proximal region of chromosome 10 was obtained with FISH and locus-specific DNA probes and with partial chromosome region-specific painting 10p11.2-10q22.2 (PCP10q-) (Figure 3a). The centromere specific D10Z1 probe provided a FISH signal at 10p11.1→q11.1 and on the sSMC. PCP10q- painted 10q10→10q23.1 on chromosome 10 and the sSMC. DNA probes specific to 10q23.1 (RP11-225E2) and to 10p14 (CELF2/BRUNOL3) (CytoCell, US) gave signals only on intact chromosome 10 (Figure 3b), showing that the breakpoints in the sSMC were located closer to the centromere than loci detected with RP11-225E2 and CELF2/BRUNOL3 (Figure 3). These data confirmed that the sSMC originated from the proximal region of chromosome 10.

**Figure 4:**
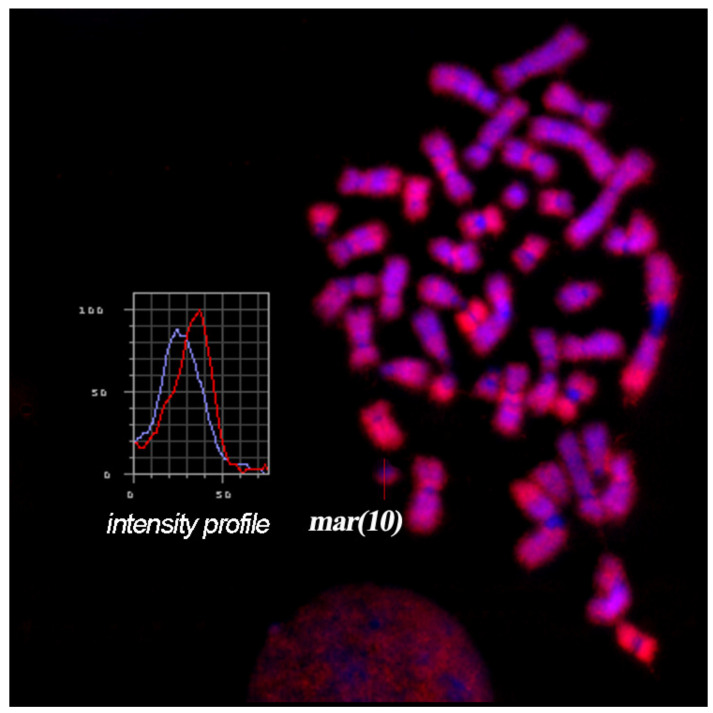
FISH with an Alu-DNA probe on foetal metaphase chromosomes. The profile of the intensity of the FISH signal and DAPI staining along the sSMC is presented in the block. Fluorescence intensity measurement along the sSMC is indicated by the red line, while the intensity of DAPI staining is shown with the blue line.

Thus, the results of CISS-hybridization with WCPmar on metaphase chromosomes of the foetus and healthy donor allowed us to conclude that the analysed sSMC is der(10) (10p11→q11.2) and contains euchromatin material from the q-arm of chromosome 10 or from its short and long arms. To confirm the presence of euchromatin region(s) in the sSMC, FISH with an Alu-DNA probe was performed. FISH provides a signal on C-negative regions of metaphase chromosomes, while it gives no FISH signal in C-positive regions (Figure 4). In the sSMC, we observed both types of regions. Part of the sSMC was painted with FISH of the Alu-DNA probe, while another part showed no FISH signal. This result is consistent with the data obtained with CISS-hybridization using WCPmar.

### 3.2. sSMC Structure Revealed with FISH Techniques

**Figure 5:**
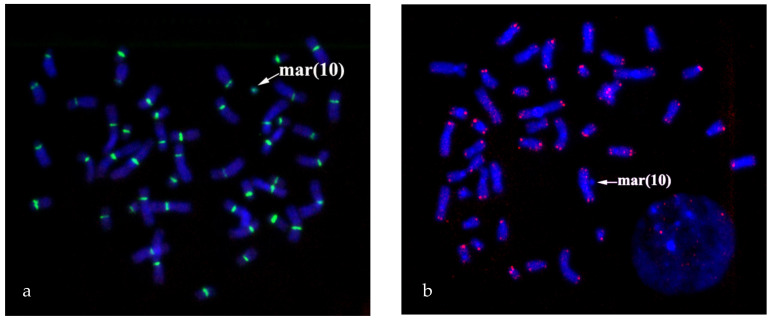
FISH analysis of the sSMC with DNA probes specific to (**a**) centromeric repeats (green signals); and (**b**) telomeric repeats (red signals). Inverted DAPI.

**Figure 6:**
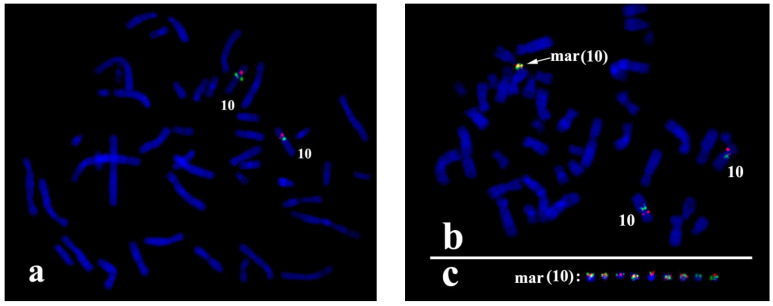
FISH with locus-specific probes *ZNF248* (10p11.21) (red signals) and *MAPK8* (10q11.22) (green signals) (**a**) on metaphase chromosomes of healthy donors; and (**b**) foetuses; (**c**) a gallery of sSMCs from different metaphase spreads with FISH signals.

Most sSMCs are derived from two-armed non-acrocentric chromosomes through two breaks in both chromosome arms. The maintenance of sSMCs during cell generation requires protection of the break sites either by the formation of new clusters of telomeric repeats or by the fusion of chromosome ends. The latter leads to a ring chromosome. The analysed sSMC contained typical centromeric repeats (Figure 5a), but no cluster of telomeric repeats was revealed in the sSMC with FISH of the labelled (TTAGGG)n repeat (Figure 5b). This result provides evidence of the ring structure of the sSMC in this case study. However, the absence of the visible FISH signal of the labelled telomeric repeat can be explained by the small size of telomeric repeat clusters on the ends of the sSMC, and additional proof of its ring nature was required. In the case of a ring chromosome, DNA fragments located near breakpoints in different arms should be located close to each other in the sSMC. In this regard, the close localization or colocalization of the distal regions of DNA from different arms of the sSMC could be considered proof of its ring structure. Two DNA probes were obtained by long-range PCR using oligonucleotide primers specific to the *ZNF248* (10p11.21) and *MAPK8* (10q11.22) genes. FISH with these DNA probes provided signals located far from each other on chromosome 10, while in the sSMC their signals were located close together (Figure 6). This result allowed us to conclude that the sSMC is a ring chromosome.

### 3.3. sSMC Content Revealed by aCGH

**Figure 7:**
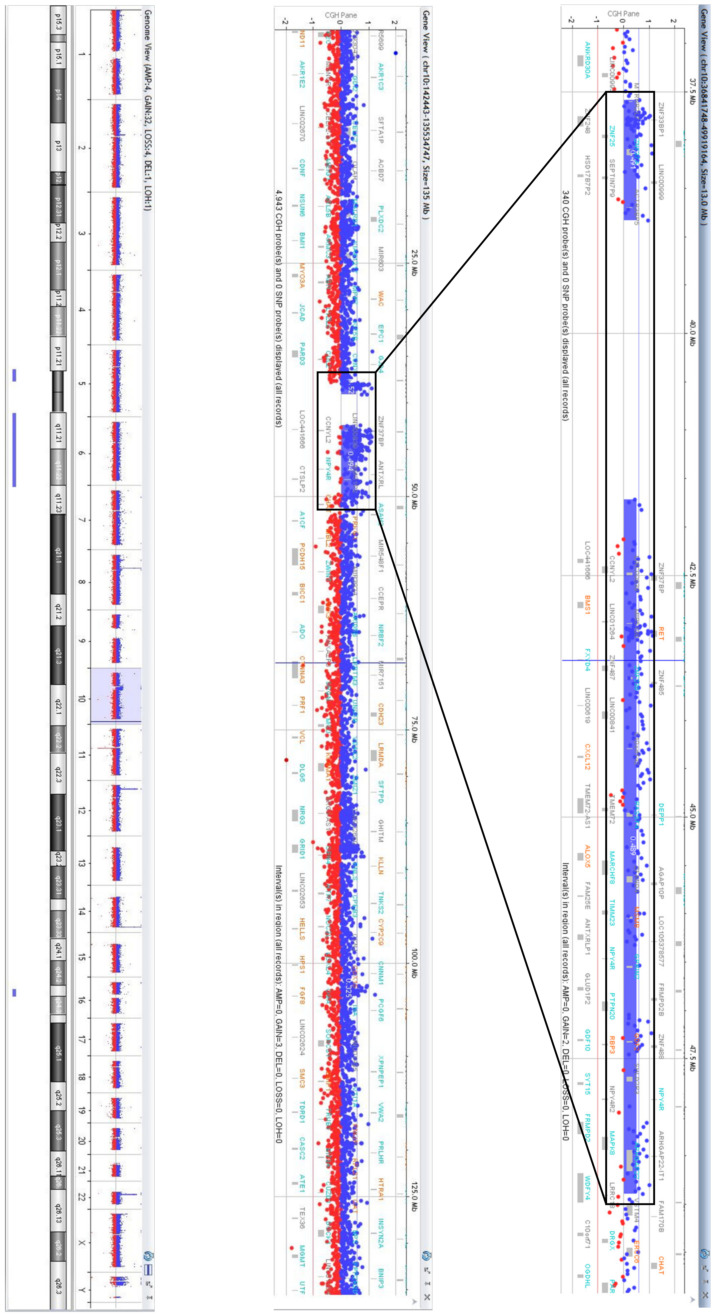
aCGH profile showing a gain on chromosome 10 in 10p11.21-p11.1 and 10q11.21-q11.23. The numbers in the axis of aCGH profiles represent log2 ratios of fluorescent intensity of tested (blue signal) and control (red signal) DNA probes. A log2-ratio of 0 corresponds to a balanced copy number; 1.0 represents the duplication.

aCGH revealed a partial trisomy 10p11.21→10q11.23. The karyotype of the foetus was 47,XX,+der(10).arr [GRCh38] 10p11.21p11.1(37588375_38831185)×3, 10q11.21q11.23 (41721019_48893792)×3 (Figure 7).

According to the Database of Genomic Variants (http://dgv.tcag.ca/dgv/app/home/, accessed on 5 May 2021), the p-arm of the sSMC encompassed 11 genes: *MTRNR2L7*, *ZNF248*, *ZNF25*, *ZNF33BP1*, *ZNF33A*, *ZNF37A*, *LOC100129055*, *HSD17B7P2*, *SEPT7P9*, *LINC00999*, and *ACTR3BP5*. None of these genes was currently referenced in the OMIM database (https://www.omim.org/, accessed on 5 May 2021) as being associated with known genetic diseases. The long arm of the sSMC contained 96 genes, among which 7 were OMIM disease-related genes (*BMS1*, *RET*, *CXCL12*, *ALOX5*, *RBP3*, *GDF2*, *MSMB*) (Table S3).

### 3.4. Confirmation of the Breakpoint Localization and Analysis of the Regions of Their Location

**Figure 8:**
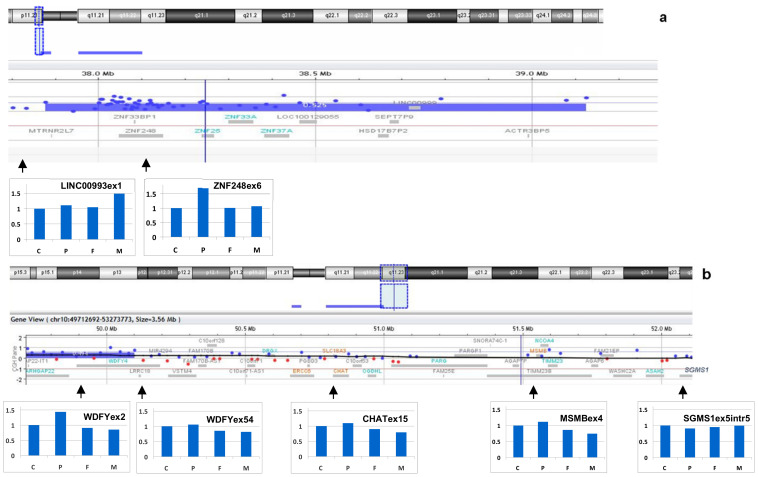
Quantitative real-time PCR analysis of the breakpoint boundaries and sSMC origin. (**a**) Confirmation of partial trisomy 10p11.21 and its boundary; (**b**) confirmation of partial trisomy 10q11 and its boundary. C—control, P—patient, F—father, M—mother.

The density of DNA sequences involved in aCGH in different parts of chromosomes is different. Usually, it is lower in the regions enriched for repetitive DNA and duplicated DNA fragments. C-positive regions, duplicon clusters, and the p-arms of acrocentric chromosomes are excluded from aCGH analysis. For confirmation of breakpoint location and analysis of the content of the regions, quantitative real-time PCR was applied to estimate the copy number for genes adjusted to the breakpoints, but located at different sites (Figure 8). Foetal DNA, parental DNA and control samples were used for this analysis. An additional copy of exon 6 of the *ZNF248* gene (located at 10p11.21 on the proximal site from the breakpoint) was registered in the foetal cells, while in the mother, father and control samples, its copy number was euploid (Figure 8). LINC00993 is located in a region from another site of this breakpoint. The same copy number was registered in the foetal, father and control samples. Unexpectedly, an additional copy of LINC00993 was registered in the mother. These data allowed us to suggest the duplication of DNA fragments in one of the mother chromosomes located very close to the breakpoint.

For analysis of the breakpoint in 10q, the copy numbers were determined for two exons of the *WDFY4* gene and for three other genes (*CHAT*, *MSMB*, and *SGMS1*). According to the aCGH results in the foetus, exons 1–43 of the *WDFY4* gene are present in three copies, while exons 44-61 are present in only two copies. We suggested that the breakpoint is at intron 43 of the *WDFY4* gene. Exon 54 of the *WDFY4* gene and the *CHAT*, *MSMB*, and *SGMS1* genes located in the region from the distal site of the breakpoint showed normal copy numbers in the foetal cells, both parental samples and control samples (Figure 8b). Exon 2 of the *WDFY4* gene showed an additional copy in the foetal cells.

The results obtained with quantitative real-time PCR were in good concordance with the conclusion made on the basis of the aCGH data. Furthermore, they indicate the de novo origin of the sSMC. Discovering the mother chromosome duplication of the DNA fragment located close to the breakpoint in 10p raised questions about the origin of the sSMC from the mother chromosome and on the possible role of this duplication in the generation of the sSMC.

### 3.5. Content of the Single Copy of the sSMC Determined by Sequencing the Microdissected DNA Library

**Figure 9:**
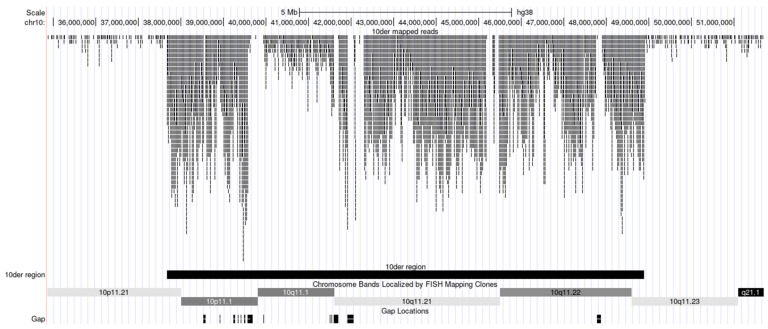
NGS read mapping for the microdissected sSMC on chromosome 10 of the hg38 reference genome. Only unique read mappings are shown.

The analysed sSMC is a ring chromosome. In cell division, break-reunion and even the loss of ring chromosomes often take place. In our study, the sSMC was observed in mosaic form. In some cells, it was lost, and we cannot exclude structural rearrangements in some cell linages. Structural rearrangements could result from the process of ring chromosome breakage in mitosis followed by the fusion of appeared ends. We hypothesize that the structural rearrangements accompanied by break-reunion events could be mostly deletions that could lead to the diversity of sSMCs in foetal cells. aCGH and quantitative real-time PCR analysis provided information on the common content of the sSMC that can be compared to that of sSMCs that differed based on various small deletions. Therefore, we performed NGS of a microdissected DNA library prepared from single copy of the sSMC (Figure 9). 

**Figure 10:**
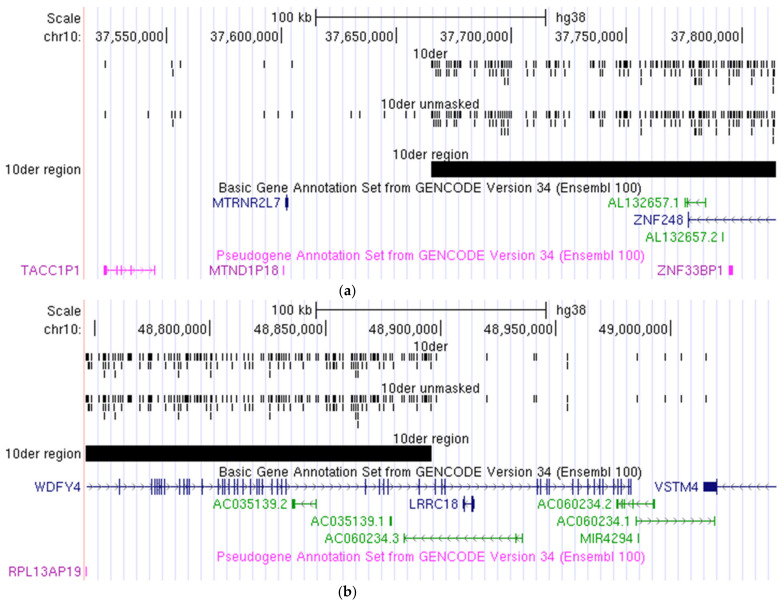
Localization of breakpoints in (**a**) 10p11.21; and (**b**) 10q11.23 in the sSMC according to NGS of the microdissected single-copy chromosome.

The hg38 reference genome was used to analyse the NGS data. We determined the borders of the sSMC as chr10:37664879-48895913 (Table 1). Furthermore, the regions of breakpoints determined by NGS of the microdissected DNA library derived from the sSMC are shown in Figure 10. 

**Figure 11:**
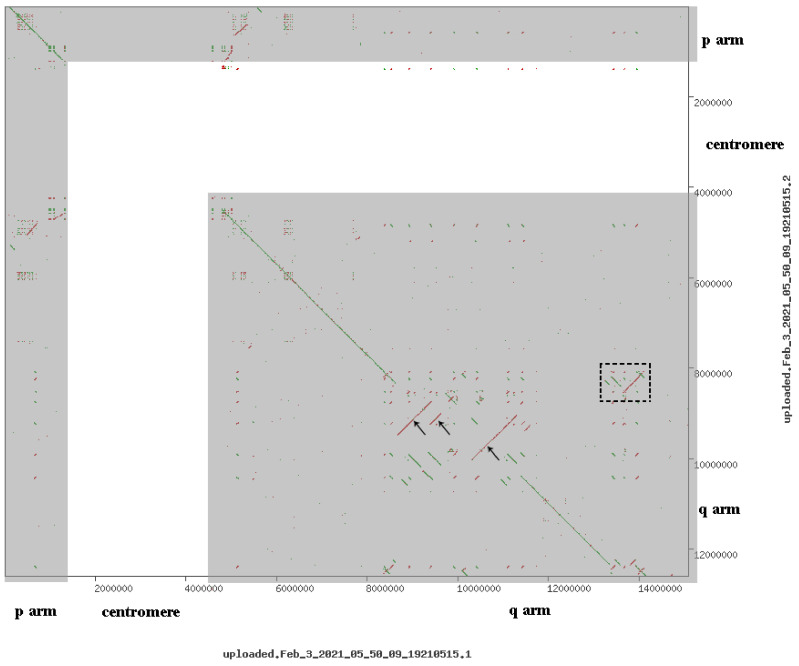
Comparative dot-plot analysis of the region of the sSMC in different human genome assemblies: hg19 (x-axis) and hg38 (y-axis). Dots indicate homologous regions. Transposable elements (SINEs, LINEs, etc.) are hard-masked with N. Dotted square to the right highlighted the area, which in the assembly hg38 is included in the additional chromosomes, but hg19 is not included in the assembly. Arrows indicate areas inversions in assemblies hg19 and hg38. p arm, centromere, q arm denote areas of the short, centromere and long arm of chromosome 10 corresponding line of coincidence of two assemblies located along diagonals. White area is a centromere region of chromosome 10, whereas grey areas denote p arm and q arm of chromosome 10.

**Figure 12:**
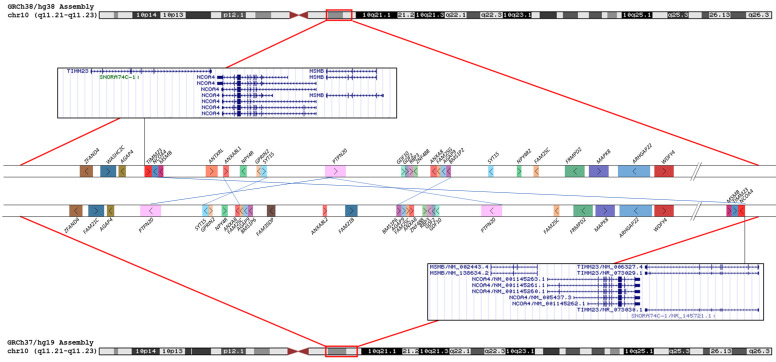
Differences in organization of the 10q11-q11.23 region between the GRCh38/hg38 and GRCh37/hg19 human genome assemblies. Blue lines denote changes in the gene’s blocks position and orientation.

Notably, there are some differences in the description of the proximal chromosome 10 region in hg19 and hg38. Their comparison revealed transposition and inversion of some regions (Figure 11 and Figure 12).

Data demonstrate that the reference genome assemblies were quite similar. A lack of similarity is characteristic of centromeric regions that are hard-masked in both assemblies. Several inversions were observed. The region with the *MSMB* gene was translocated in hg19 relative to hg38. As a result, it is located outside of the region involved in sSMC formation according to the more recent version of the genome assembly.

What is the reason for this difference? It is possible that some differences are the result of correction, while others could be derived from structural differences between individual genomes involved in human reference genome sequencing. This complicates the direct comparison of the results obtained by aCGH and NGS of the microdissected DNA library. However, the content of the sSMC revealed with different techniques appeared to be very similar. In 10p11.21, the breakpoint was assigned between genes *MTRNR2L7* and *ZNF248* (Figure 10a), while in 10q11.23, it was localized within intron 43 of *WDFY4* (Figure 10b), which was also confirmed by real-time PCR analyses.

However, in proximal regions of chromosome 10 corresponding to the sSMC, there were small regions showing no homology to the reads from the microdissected library (Figure 9). We cannot exclude that these gaps are just technique artefacts, but they are probably trace of the events of ring chromosome breakage followed by the fusion of appeared ends. The breakage of the ring chromosome taking place in mitosis can accompany degradation of unprotected chromosome ends before their fusion. One deletion was rather large. Twenty-two genes are known to be located in the deleted region (Table S4). If this is true, some individual ring sSMCs may have another size and gene content than described with aCGH based on analysis of the bulk chromosome copies.

## 4. Discussion

**Figure 13:**
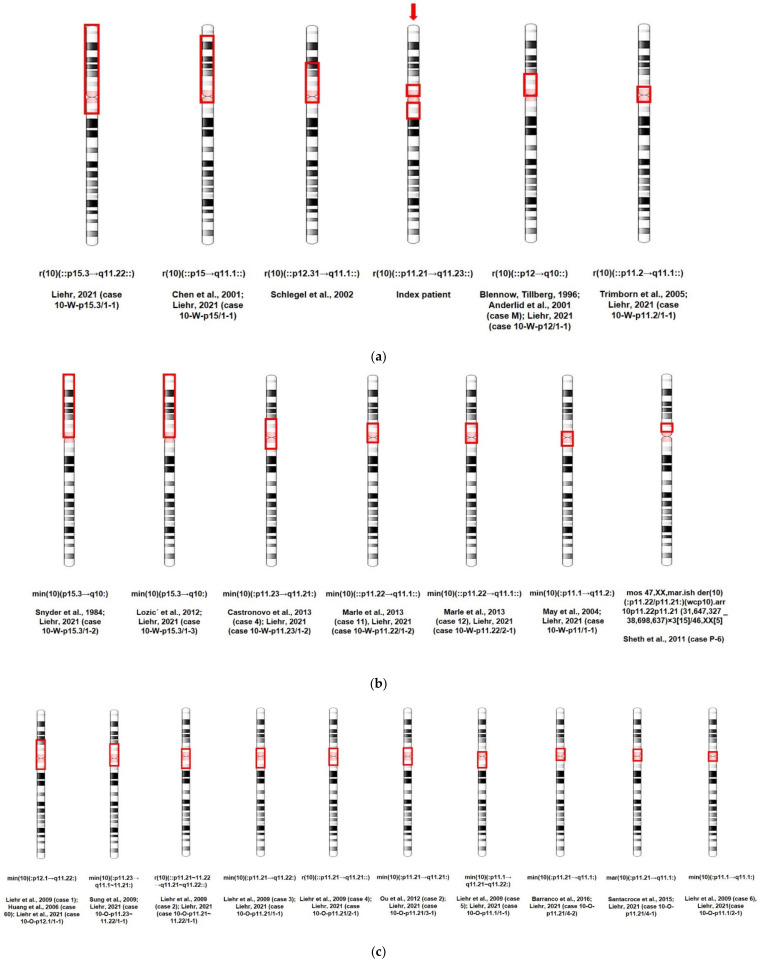
The size and origin of sSMC(10)s. The affected regions outlined by the red rectangle on chromosome 10 in the index patient are marked by red arrows. (**a**) Patients with supernumerary ring chromosome 10; (**b**) clinically affected carriers of an sSMC(10); (**c**) healthy carriers of an sSMC(10). The references are given in the Supplementary Materials.

To the best of our knowledge, five cases of supernumerary ring chromosome 10 have been reported in the literature (Table S5, Figure 13a). In two cases, the ring included almost the whole p-arm of chromosome 10 (#1, #2). In other patients, the rings were smaller, and the smallest ring was in proband #6. Patients #1–3 and #5 exhibited developmental delay, intellectual disability, speech and language delay, skull and skeletal abnormalities, and facial dysmorphia. Patient #6, a girl with the smallest reported r(10), had a normal birth length and weight (51 cm (75th percentile) and 3120 g (25–50th percentile), respectively). However, at the age of 12 years, her length was 132.5 cm, and her weight was 25.8 kg, both below the 3rd centile. Her skeletal age was delayed by approximately two years, and she did not show signs of puberty. The girl had normal intelligence, but showed attention deficit disorder. Our patient (#4) at 11 weeks of gestation had a slightly elevated level of ß-HCG, and at 20.4 weeks of gestation, hypoplasia of the nasal bone became the indications for invasive prenatal diagnosis. The girl was healthy at birth and was healthy at 1 year and 1 month of age. Her weight was 9.8 kg (25–50th percentile), and her height was 74 cm (25–50th percentile).

To date, seven cases of sSMC(10) not specifically examined for ring structure have been described in patients with abnormal clinical signs (Table S6, Figure 13b). Two markers consisted of the whole short arm of chromosome 10—patients #1 and #2 in Table S5. Both probands were characterized by intrauterine growth restriction and oligohydramnios in the prenatal period, low birth weight (2280 g and 2400 g, respectively), skull abnormalities, multiple facial dysmorphisms, hypotonia, and heart defects. Patients #3–7 with much smaller markers had less severe phenotypic abnormalities (Table S6).

Data on individuals with sSMC(10) also not specifically examined for ring structure and without clinical abnormalities are summarized in Table S7 and Figure 13c. Cases #1–5 and #7–10 were diagnosed prenatally: #1 and 7—due to abnormal screening and #2, #3, and #10—due to advanced maternal age. The babies were apparently healthy at birth. Patients #1, #4, and #10 were followed up to 1, 10, and 2 years old and were healthy. A different case is patient #9—a 40-year-old phenotypically normal woman with long-term infertility and 47,XX,+mar [49]/46,XX [51] karyotype. sSMC(10) was also detected in her apparently healthy mother and sister: 47,XX,+mar [61]/46,XX [39] and 47,XX,+mar [77]/46,XX [23], respectively.

It is worth noting that for patients with markers (Tables S6 and S7), FISH with telomere-specific DNA probes was not performed, i.e., the ring form of the marker chromosome could not be excluded. In our patient (# 4 in Table 1), the ring form of sSMC(10) was confirmed by FISH with telomere-specific DNA probes and locus-specific identifiers for distal sequences of sSMC—*ZNF248* (10p11.21) and *MAPK8* (10q11.22). Interestingly, there was a sex bias with female prevalence in the sex ratio among the patients with confirmed r(10) and sSMC(10)—1:5 and 2:17, respectively.

It seems that our patient has the longest q-arm of sSMC(10) reported in the literature without any visible clinical consequences at least at 1 year and 1 month of age (Figure 13a). Despite a detailed analysis of the structure and content of the sSMC and its probable presence in most foetal cells, genetic counselling presented serious problems in our case. It was previously shown that marker chromosomes containing up to 3–5 Mb of euchromatin material of pericentromeric origin may not lead to phenotypical consequences for carriers [19]. The excess euchromatin in our patient was much larger and exceeded 8.9 Mb. Several hypotheses should be discussed. The results of the single-copy chromosome sequencing in our study indicate that each individual supernumerary marker chromosome may contain less euchromatin material due to deletions in its structure. Thus, the carrier of such a marker chromosome is a mosaic for a copy number of genes.

Significantly, deletion of some genes in the supernumerary marker chromosomes took place in some cells, leading to a normal copy number of these genes in the cells from the clones derived from them. In addition, a decrease in the size of euchromatin regions may be of fundamental importance. The nuclear periphery is predominantly occupied by transcriptionally inactive chromatin, such as centromeres and regions of constitutive and facultative heterochromatin. It is possible that the whole chromosome arm promotes the removal of the proximal region of the chromosome into the compartment of transcriptionally active chromatin, while a small region of euchromatin in the sSMC may remain at the periphery of the nucleus in the transcriptionally inactive compartment. Thus, the pathogenetic significance of the marker chromosome may be reduced due to this feature of its euchromatin localization in the interphase nucleus. This decrease in the pathogenetic potential can be facilitated by both a decrease in the sSMC size and its ring organization. In the presented sSMC case, both of these factors are likely to be present: the ring organization of the sSMC was confirmed by FISH analyses with telomere-specific DNA probes and unique locus-specific identifiers, whereas the NGS data of the microdissected DNA library from a single copy of the sSMC suggest the presence of various small deletions.

Data obtained indicate that the ring nature of the sSMC should be considered separately during genetic counselling, taking into account the possibility of ring chromosome secondary rearrangements and inherent instability [20]. The precise determination of the localization of the breakpoint during formation of the ring sSMC does not always allow us to accurately describe its composition and changes in structure and gene content between different cell clones due to dynamic mosaicism. It should also be noted that the appearance of small deletions in ring sSMCs can lead to disruption of the topologically associated domains (TADs) in the 3D interphase nuclei structure, which can alter the transcription regulation mechanisms. Despite significant advances in the study of additional chromosomal elements of the genome, the exact mechanism of their origin is not clear. It is difficult to unequivocally answer at what stage of cell division the formation of the sSMC took place, but most likely it is derived from the maternal chromosome 10.

Unfortunately, even such a comprehensive study of the marker chromosome did not allow a reliable prediction of its effect on the carrier phenotype. The family decided to prolong the pregnancy after cordocentesis and genetic counselling. As a result, a healthy girl with no visible developmental anomalies was born at term. At the same time, the genetic risks of the sSMC manifestation should be taken into account and carefully controlled during her life by clinical geneticists.

## 5. Conclusions

Based on our results, it is possible to recommend the following algorithm for the molecular diagnosis of sSMCs:

1. Detection of sSMCs during routine cytogenetic analysis of G-banded patient metaphase chromosomes;
2. Determination of the chromosome-derived origin of the revealed sSMC and preliminary assessment of its composition by chromosomal microdissection and CISS-hybridization with WCPmar;
3. Control of the presence of euchromatin material in the sSMC using FISH with labelled Alu repeats on the patient’s metaphase chromosomes;
4. Analysis of mosaicism by the sSMC by CISS-hybridization on interphase nuclei;
5. Determination of the breakpoint localization and gene content of the sSMC by aCGH if euchromatin is detected in the sSMC and the level of mosaicism is higher than 15–20%;
6. Analysis of the structural organization of the sSMC by FISH with a labelled DNA probe specific to telomeric repeats;
7. Design of DNA probes specific to the distal loci at each end of the sSMC in the absence of the FISH signal on the target telomeric repeats and using these probes to perform two-colour FISH;
8. Taking into account the possibility of a decrease in the size of euchromatin regions in different copies in the case of sSMCs due to dynamic mosaicism in ring chromosomes;
9. Testing for uniparental disomy, if the sSMC derived from human chromosomes with known genomic imprinting (namely, chromosomes 6, 7, 8, 11, 14, 15, 19, and 20).

## Figures and Tables

**Table 1:** Break-point coordinates in the sSMC(10) detected by aCGH and single-copy chromosome sequencing.

	aCGH	Microdissected sSMC Sequencing
Start	37,588,375	37,664,879
End	48,893,792	48,895,913
Size	11.305.417 bp	11.231.034 bp

## Data Availability

The data that support the findings of this study are available from the corresponding author upon reasonable request.

## Supplementary Materials

The following are available online at https://www.mdpi.com/article/10.3390/biomedicines9081030/s1, Table S1: Primers for amplification of the unique sequences of genes *ZNF248* (10p11.21) and *MAPK8* (10q11.22) by long-range PCR on the sSMC and chromosome 10, Table S2: Primer sequences for real-time PCR, Table S3: OMIM genes in the long arm of the sSMC(10), Table S4: The 22 genes located in the deleted region within the microdissected single-copy sSMC(10), Table S5: Clinical symptoms in patients with supernumerary ring chromosome 10, Table S6: Symptoms in patients with small supernumerary marker chromosomes originating from chromosome 10, Table S7: Patients with small supernumerary marker chromosomes originating from chromosome 10 and no clinical signs.

## References

[1] Liehr T. (2012). Small Supernumerary Marker Chromosomes (sSMC).

[2] Jafari-Ghahfarokhi H., Moradi-Chaleshtori M., Liehr T., Hashemzadeh-Chaleshtori M., Teimori H., Ghasemi-Dehkordi P. (2015). Small supernumerary marker chromosomes and their correlation with specific syndromes. Adv. Biomed. Res..

[3] Liehr T., Ewers E., Kosyakova N., Klaschka V., Rietz F., Wagner R., Weise A. (2009). Handling small supernumerary marker chromosomes in prenatal diagnostics. Expert Rev. Mol. Diagn..

[4] Al-Rikabi A.B.H., Pekova S., Fan X., Jančušková T., Liehr T. (2018). Small supernumerary marker chromosome may provide information on dosage-insensitive pericentric regions in human. Curr. Genom..

[5] Liehr T., Al-Rikabi A. (2019). Mosaicism: Reason for normal phenotypes in carriers of small supernumerary marker chromosomes with known adverse outcome. A systematic review. Front. Genet..

[6] Seabright M. (1971). A rapid banding technique for human chromosomes. Lancet.

[7] Henegariu O., Heerema N.A., Lowe Wright L., Bray-Ward P., Ward D.C., Vance G.H. (2001). Improvements in cytogenetic slide preparation: Controlled chromosome spreading, chemical aging and gradual denaturing. Cytometry.

[8] Rubtsov N.B., Karamisheva T.V., Astakhova N.M., Liehr T., Claussen U., Zhdanova N.S. (2000). Zoo-FISH with region-specific paints for mink chromosome 5q: Delineation of inter- and intrachromosomal rearrangements in human, pig, and fox. Cytogenet. Genome Res..

[9] Karamysheva T.B., Matveeva V.G., Shorina A.P., Rubtsov N.B. (2001). Clinical and molecular cytogenetic analysis of a rare case of mosaicism for partial trisomy 3p and partial trisomy 10q in humans. Russ. J. Genet..

[10] Lichter P., Cremer T., Borden J., Manuelidis L., Ward D.C. (1988). Delineation of individual human chromosomes in metaphase and interphase cells by in situ suppression hybridization using recombinant DNA libraries. Hum. Genet..

[11] Ijdo J.W., Wells R.A., Baldini A., Reeders S.T. (1991). Improved telomere detection using a telomere repeat probe (TTAGGG)n generated by PCR. Nucleic Acids Res..

[12] Zhigalina D.I., Skryabin N.A., Vasilieva O.Y., Lopatkina M.E., Vasiliev S.A., Sivokha V.M., Belyaeva E.O., Savchenko R.R., Nazarenko L.P., Lebedev I.N. (2020). FISH diagnostics of chromosomal translocation with the technology of synthesis of locus-specific DNA probes based on long-range PCR. Russ. J. Genet..

[13] Batzer M.A., Deininger P.L. (1991). A human-specific subfamily of Alu sequences. Genomics.

[14] Makunin A.I., Kichigin I.G., Larkin D.M., O’Brien P.C.M., Ferguson-Smith M.A., Yang F., Proskuryakova A.A., Vorobieva N.V., Chernyaeva E.N., O’Brien S.J. (2016). Contrasting origin of B chromosomes in two cervids (Siberian roe deer and grey brocket deer) unravelled by chromosome-specific DNA sequencing. BMC Genom..

[15] Martin M. (2011). Cutadapt removes adapter sequences from high-throughput sequencing reads. EMB Net. J..

[16] Li H. (2013). Aligning sequence reads, clone sequences and assembly contigs with BWA-MEM. arXiv.

[17] Seshan V.E., Olshen A. DNAcopy: DNA Copy Number Data Analysis; R Package Version 1.66.0; Bioconductor: 2021. https://bioconductor.org/packages/release/bioc/html/DNAcopy.html.

[18] Untergasser A., Cutcutache I., Koressaar T., Ye J., Faircloth B.C., Remm M., Rozen S.G. (2012). Primer3—New capabilities and interfaces. Nucleic Acids Res..

[19] Liehr T. (2014). Small supernumerary marker chromosomes–an update. Mol. Cytogenet..

[20] Nikitina T.V., Kashevarova A.A., Gridina M.M., Lopatkina M.E., Khabarova A.A., Yakovleva Y.S., Menzorov A.G., Minina Y.A., Pristyazhnyuk I.E., Vasilyev S.A. (2021). Complex biology of constitutional ring chromosomes structure and (in)stability revealed by somatic cell reprogramming. Sci. Rep..

