# Supplementary material for: Prenatal Diagnosis of Small Supernumerary Marker Chromosome 10 by Array-Based Comparative Genomic Hybridization and Microdissected Chromosome Sequencing
Source: Biomedicines. 2021 Aug 17;9(8):1030. doi: 10.3390/biomedicines9081030 (PMC8391546; doi:10.3390/biomedicines9081030)
Supplement: Supplementary file 1 [file biomedicines-09-01030-s001.zip › biomedicines-1269011-supplementary.pdf]

biomedicines

MDPI

Supplementary Materials

Table S1. Primers for amplification of the unique sequences of genes *ZNF248* (10p11.21) and *MAPK8* (10q11.22) by long-range PCR on the sSMC and chromosome 10.

	Primer	Sequence	Tm, °C	Coordinate(hg19)	Lengths, bp
1	ZNF248_FISH1-F	CCTTGGGTATCCTTGATGCCTTC	61	chr10:38107676-38116724	9048
	ZNF248_FISH1-R	TTGGTAGGCCAAACATCGTCAAA	61		
2	ZNF248_FISH2-F	GTATAGCAGCACAGAGGGAGGTA	61	chr10:38119281-38127164	7883
	ZNF248_FISH2-R	ACAGTCAGAGGAAAGGTACGTGA	61		
3	ZNF248_FISH3-F	TTACAGAGCAGAACTCAGTGCAT	60	chr10:38137765-38144931	7166
	ZNF248_FISH3-R	TAGAGGACACACTTCTCAACAGC	60		
4	ZNF248_FISH5-F	GGTGGGAACTTGACTGTTGTCAT	61	chr10:38070656-38079918	9263
	ZNF248_FISH5-R	ACATTGACCAGGCACTGACATTT	61		
5	ZNF248_FISH6-F	AAATGTCAGTGCCTGGTCAATGT	61	chr10:38061754-38070678	8924
	ZNF248_FISH6-R	AACAGCGTTCCACATTTCTGACT	61		
6	ZNF248_FISH7-F	GAGTGTAAGCTGGCATCCTAACC	61	chr10:38079896-38088572	8676
	ZNF248_FISH7-R	ATGACAACAGTCAAGTTCCCACC	61		
7	MAPK8_FISH1-F	CCGATATCACGAGCAGGTTTCTA	60	chr10:49638588-49646916	8328
	MAPK8_FISH1-R	GAACAGTTGTTGCAGTGGTCTAC	60		
8	MAPK8_FISH2-F	TGTTCAGAGATAAACGGCCAAGA	60	chr10:49607326-49614847	7521
	MAPK8_FISH2-R	CGTAGCATGGTGCTTAAATCAGG	60		
9	MAPK8_FISH3-F	CATGGTGTTCCAAGCTGTTCAAT	60	chr10:49625366-49633976	8610
	MAPK8_FISH3-R	GGCTGTCTTCAGTCTTCCTGTAA	60		
10	MAPK8_FISH4-F	AACTAGTCTTTGGCTCTACCTGC	60	chr10:49612629-49620784	8155
	MAPK8_FISH4-R	GATCTTTGAGGCAACACAACGAA	60		
11	MAPK8_FISH5-F	CATGTGGTGGGCAATAACGTATC	60	chr10:49575946-49585684	9738
	MAPK8_FISH5-R	CATTCAACTGGATGGGCTTTGTT	60		
12	MAPK8_FISH7-F	ACACAAAGATGACTCCCTGATCC	60	chr10:49562806-49570496	7690
	MAPK8_FISH7-R	CAGTGCGAATTTAACGCTGTGTA	60		

Table S2. Primer sequences for real-time PCR

Region	Primer	Sequence, 5′ → 3′
5q13	HEXB F	CCGGGCACAATAGTTGAAGT
	HEXB R	TCCTCCAATCTTGTCCATAGC
10q11.21q11.23	WDFY4ex2 F	AGACAGAAATGAAGACCCAGGT
	WDFY4ex2 R	GCTCTTCTGACGCTCAATGG
10q11.21q11.23	WDFY4ex54 F	ATCTTCCACCCCTACTTCTACG
	WDFY4ex54 R	CACCTGTCCAAAGTTGCTGAC
10q11.21q11.23	CHATex15 F	AAACACATTTCTGCTTTCGTGC
	CHATex15 R	GGCTGTGCAGTTTACTCATCTT
10q11.21q11.23	MSMBex4 F	TGACAAAGACAACTGCCAAAGA
	MSMBex4 R	TTAGAGGCCAGAGGAGAATGAG
10q11.21q11.23	SGMS1ex5intr5 F	GAAAGTGTCTGGTTGGGAAACA
	SGMS1ex5intr5 R	CAATACATACCTGCAACAGCCA
10p11.21	LINC00993ex1 F	GTTGAAGAGATGAGTGCGGG
	LINC00993ex1 R	TTTACTGGGAGGTGGCAAGAG
10p11.21	ZNF248ex6 F	AAAGTGAGTGACAAAACCTGGG
	ZNF248ex6 R	CTCTCCGAAGCTGGGTAAGAT

Table S3. OMIM genes in the long arm of the sSMC(10).

Gene symbol	Gene full name	Gene MIM number	Gene-phenotype relationships	Phenotype MIM number	Related biological processes/pathological phenotype
BMS1	BMS1 ribosome biogenesis factor	611448	Nonsyndromic aplasia cutis congenita	107600	Liver abnormalities, intestinal malrotation [21].
RET	Ret proto-oncogene	164761	Susceptibility to Hirschsprung disease	142623	Parkinson’s disease, retinitis pigmentosa, neuropathic pain, obesity and overweight, carcinogenesis [22].
			Protection against Hirschsprung disease		
			Central hypoventilation syndrome	209880	
			Medullary thyroid carcinoma	155240	
			Multiple endocrine neoplasia IIA	171400	
			Multiple endocrine neoplasia IIB	162300	
			Pheochromocytoma	171300	
CXCL12	C-X-C motif chemokine ligand 12	600835	Resistance to AIDS	609423	Embryogenesis, haematopoiesis angiogenesis, carcinogenesis [23], muscle regeneration [24]. Atherosclerosis, coronary artery disease [25], viral infections, neurodegenerative diseases, inflammatory bowel diseases, asthma and acute lung injury, rheumatoid arthritis, ocular diseases, diabetic retinopathy [23], polycystic kidney disease [26].
ALOX5	Arachidonate 5-lipoxygenase	152390	Diminished response to antileukotriene treatment in asthma	600807	Atherosclerosis [27], Alzheimer's disease [28], chronic myeloid leukaemia [29].
RBP3	Retinol binding protein 3	180290	Retinitis pigmentosa 66	615233	High myopia, retinal dystrophy [30], diabetic retinopathy [31].
GDF2	Growth differentiation factor 2	605120	Hereditary haemorrhagic telangiectasia, type 5	615506	Glucose homeostasis [32], osteogenesis [33], adipogenesis [34], cholinergic neurons differentiation [35], carcinogenesis [36], coronary heart disease, hypertension [21], rheumatoid arthritis [37].
MSMB	Microseminoprotein beta	157145	Hereditary prostate cancer	611928	Allergic rhinitis [38].

Table S4. The 22 genes located in the deleted region within the microdissected single-copy sSMC(10)

Name	Minimum	Maximum	Length	Direction
WDFY4	48866907	48982957	>116050	forward
LRRC18	48909481	48940127	30647	reverse
LOC105378298	48976963	48981359	4397	reverse
MIR4294	48985513	48985588	76	reverse
VSTM4	49014237	49129119	114883	reverse
FAM170B-AS1	49121840	49151548	29709	forward
FAM170B	49131155	49134022	2868	reverse
TMEM273	49154729	49188581	33853	reverse
C10orf71-AS1	49296284	49298894	2611	reverse
C10orf71	49297010	49330146	33137	forward
DRGX	49364067	49396090	32024	reverse
ERCC6	49434882	49539539	104658	reverse
HSPD1P17	49440817	49440957	141	forward
PGBD3	49515106	49524282	9177	reverse
HMGB1P50	49551309	49552653	1345	forward
CHAT	49609096	49667943	58848	forward
SLC18A3	49610311	49612721	2411	forward
C10orf53	49679652	49710262	30611	forward
OGDHL	49734642	49762380	27739	reverse
MAPK6P6	49771064	49774419	3356	forward
RPL21P89	49815059	49815599	541	forward
PARG	49818275	49897925	>79651	reverse

Table S5. Clinical symptoms in patients with supernumerary ring chromosome 10

Case №	GTG-banding results	Additional tests, methods	Studied material	*De novo*/ inherited	Sex/ age at diagnosis	Clinical symptoms	Literature, ChromoSomics Database [39]
1	47,XX,+mar[9]/46,XX[56]<br><br>r(10)(::p15.3→q11.22::)<br><br>mos47,XX,+r(10) (::p15.3→q11.22::)[9]/ 46,XX[56]	FISH with centro-mere-specific probe, sub-cen M-FISH, MCB	PBL	n.a.	female/ 10 y.o.	1. Mild intrauterine growth retardation in the last trimester;<br>2. Feeding problems after birth;<br>3. Bilateral talipes equinovarus;<br>4. Slightly different facial features;<br>5. Slightly flat midface and rather prominent large ears;<br>6. Intellectual impairment	Case 10-W-p15.3/1-1 [39]
2	47,XX,+mar[7]/46,XX[13]<br><br>r(10)(::p15→q11.1::)<br><br>mos47,XX,+r(10) (::p15→q11.1::)[7]/ 46,XX[13]	WCP, SKY	PBL	*de novo*	female/ 9 m	1. Born at 39 weeks of gestation;<br>2. Birth weight 2951 g;<br>3. Hyperextension;<br>4. Dislocation of the knees and ocular hyper-telorism at birth;<br>5. Dolichocephaly;<br>6. Prominent forehead at nasal root;<br>7. Prominent cheeks;<br>8. Down-turned corners of the mouth;<br>9. Micrognathia;<br>10. Hypotonia;<br>11. Global developmental delay	Case 10-W-p15/1-1 [39,40]
3	Short-term culture 47,XY,+mar[15]/46,XY[2]<br><br>Long-term culture 47,XY,+mar[9]/ 46,XY[2]<br><br>Fibroblast culture 47,XX,+mar[4]/	FISH using the micro-dissected chromosome library, UPD-test	CVS/AF /F	*de novo*	female/ prenatal	1. CVS was performed at 13 weeks of gesta-tion;<br>2. Ultrasound revealed a twin pregnancy with one twin smaller and without cardiac activ-ity;<br>3. Growth parameters of the surviving twin corresponded to 12 and 5/7 weeks of gesta-tion;<br>4. The pregnancy was terminated at 18 and 2/7	[41]

	46,XX[6]<br><br>r(10)(::p12.31→ q11.1::) 88%/CVST, 81%/CVLT, 50%/AFC, 40%/F<br><br>mos47,XY,+r(10)(::p12.31 →q11.1::)[15]/46,XY[2]					weeks;<br>5. The foetus weighed 180 g with a crown–rump length<br>6. (CRL) of 20 cm.<br>7. Swelling of the neck;<br>8. Hypoplasia of heart, liver, kidneys and suprarenal glands;<br>9. Double ureter;<br>10. matUPD10.	
4	47,XX,+r(10) (::p11.21→ q11.23::)	CISS with WCPmar probe, aCGH, NGS of single-copy microdissected library, real-time PCR	Cord blood	*de novo*	female/ prenatal	1. At 11 weeks of gestation a slightly elevated level of ß-HCG;<br>2. At the 20.4 weeks of gestation hypoplasia of nasal bone	Index patient
5	47,XY,+r[19]/ 46,XY[10]<br><br>r(10)(::p12→q10::)<br><br>mos47,XY,+r(10)(::p12→ q10::)[19]/46,XY[10]	FISH, UPD-test	PBL	n.a.	male/ 15 y.o.	1. Born after 41 weeks of gestation;<br>2. Birth weight - 2300 g and a birth length - 48 cm;<br>3. At 15 years of age height was 153 cm (-3 SD) and head circumference - 51 cm (-2 SD);<br>4. Unilateral cleft lip and palate;<br>5. Low set ears with a prominent anthelix;<br>6. Micrognathia;<br>7. The eyes had a mild upward slant;<br>8. General muscular weakness with predominantly upper limb hypotonia;<br>9. Mild mental retardation with an IQ of approximately 70	Case 10-W-p12/1-1 [39,42]

6	47,XX,+mar[8]/ 46,XX[48] in PBL (in OM sSMC in 16%)<br><br>r(10) (::p11.2→q11.1::)<br><br>mos47,XX,+r(10)(::p11.2 →q11.1::)[8]/46,XX[48]	WCP, FISH with centromere-specific probe, UPD-test	PBL/oral mucosa	*de novo*	female/ 12 y.o	1. Birth length - 51 cm and weight - 3120 g;<br>2. At 6 months thrombocytopenia;<br>3. At 12 y height was 132.5 cm, weight - 25.8 kg, skeletal age ~2 y delayed;<br>4. No signs of puberty;<br>5. IGF1 levels decreased;<br>6. Normal intelligence, but attention deficit disorder	Case 10-W-p11.2/1-1 [39,43]

Footnote for Tables S5-S7. aCGH – array-based comparative genomic hybridization; AF – amniotic fluid; AFC - amniotic fluid culture; CISS - chromosomal *in situ* suppression; CVLT - chorionic villus long term; CVS - chorionic villus sampling; CVST - chorionic villus short term; F – fibroblasts; FISH – fluorescence *in situ* hybridization; IVF – *in vitro* fertilization; MCB – multicolour banding; n.a. – not available; OM – oral mucosa; PBL – peripheral blood lymphocytes; SKY - spectral karyotyping; UPD – uniparental disomy; WCP - whole-chromosome painting.

Table S6. Symptoms in patients with small supernumerary marker chromosomes originating from chromosome 10

Case №	GTG-banding results	Additional tests, methods	Studied material	*De novo*/ inherited	Sex/ age at diagnosis	Clinical symptoms	Literature, ChromoSomics Database
1	47,XX,+mar<br><br>min(10)(p15.3→q10:)<br><br>47,XX,+10p(pter→cen)<br><br>47,XX,+min(10)<br>(:p15.3→q10:)	Not performed	PBL/F	*de novo*	female/<br>1 y.o.	1. Born at 37 weeks gestation;<br>2. The pregnancy was complicated by vaginal bleeding at 5 months gestation;<br>3. Intrauterine growth retardation with oligohydramnios in the third trimester;<br>4. Apgar scores at delivery were 2 at one minute and 6 at five minutes;<br>5. A two vessel cord;<br>6. Birth weight 2280 g, length 48 cm, and head circumference 30.5 cm;<br>7. Hypotonia;<br>8. Ventricular septal defect;<br>9. Right sided renal agenesis;<br>10. Dolichocephaly, wide sutures, upswept frontal hair-pattern, broad nasal root, inverted boarder of the lips;<br>11. Short neck;<br>12. Hypoplastic nails;<br>13. Bilateral talipes equinovarus	Case<br>10-W-p15.3/1-2<br>[39,44]
2	47,XX,+mar<br><br>min(10)(p15.3→q10:)<br><br>47,XX,+min(10)<br>(:p15.3→q10:)	WCP, FISH with a probe specific for the centromeric region of chromosome 10, a probe for the locus at region 10p14	PBL	mat	female/<br>1 y.o.	1. Intrauterine growth restriction and oligohydramnios;<br>2. Birth weight 2400 g, length 47 cm, head circumference 33 cm;<br>3. Dolichocephaly with wide sutures/fontanelles, elongated face, high frontal hairline, hypertelorism, large ears, bilateral cleft lip and palate, apparently wide and flat nasal bridge, prominent cheeks;<br>4. At 4 months: microcephaly, barrel shaped	Case<br>10-W-p15.3/1-3<br>[39,45]

						chest, marbled skin, atrial septal defect, flexion contractures of large joints, clubfoot anomaly;<br>5. Generalized hypotonia;<br>6. Growth and developmental delay	
3	47,XX,+mar[3]/<br>46,XX[7]<br><br>min(10)(:p11.23→<br>q11.21:)<br><br>mos47,XX,<br>+min(10)(:p11.23→q11.2<br>1:)[3]/46,XX[7]	Pericentric BAC-set	AF	*de novo*	female/<br>prenatal	1. Advanced maternal age;<br>2. Pre-term delivery;<br>3. Failure to thrive;<br>4. Dysmorphism;<br>5. Neonatal hypotonia	Case<br>10-W-p11.23/1-2<br>[39];<br>Case 4 [46]
4	47,XY,+mar[5]/46,XY[19]<br><br>min(10)(::p11.22→<br>q11.1::)<br><br>mos47,XY,+min(10)<br>(::p11.22→q11.1::)[5]/<br>46,XY[19]	aCGH	AF	n.a.	male/<br>prenatal	1. Advanced maternal age;<br>2. Born with tetralogy of Fallot	Case 10-W-<br>p11.22/1-2) [39];<br>Case 11 [47]
5	47,XX,+mar[19]/<br>46,XX[56]<br><br>min(10)<br>(::p11.22→q11.1::)<br><br>mos47,XX,+min<br>(10)(::p11.22→<br>q11.1::)[19]/46,XX[56]	aCGH	AF	n.a.	female/<br>prenatal	Horseshoe kidney	Case<br>10-W-p11.22/<br>2-1 [39];<br>Case 12 [47]

6	47,XX,+mar[24]/ 46,XX[26] in PBL (in AF 13/15)<br><br>min(10)(:p11.1→q11.2:)<br><br>mos47,XX,+min (10)(:p11.1→q11.2:)[24]/ 46,XX[26]	Subcen M-FISH	AF/PBL	*de novo*	female/ prenatal	1. At 1 y.o. height 90-95th centile;<br>2. At 2 y.o. oral dyspraxia	Case 10-W-p11/1-1 [39,48]
7	mos 47,XX,mar.ish der(10) (:p11.22/p11.21:)(wcp10) .arr 10p11.22p11.21 (31,647,327 _ 38,698,637)×3[15]/ 46,XX[5]	aCGH	PBL	*de novo*	female/ 1 y.o.	1. Urogenital abnormalities;<br>2. Left hypoplastic kidney;<br>3. Vaginal atresia	Case P-6 [49]

Table S7. Patients with small supernumerary marker chromosomes originating from chromosome 10 and no clinical signs

Case №	GTG-banding results	Additional tests, methods	Studied material	*De novo*/ inherited	Sex/ age at diagnosis	Clinical symptoms	Literature, ChromoSomics Database
1	47,XX,+mar[7]/ 46,XX[8]<br><br>min(10)(:p12.1→ q11.22:)<br><br>mos47,XX,+min (10)(:p12.1 →q11.22:)[7]/ 46,XX[8]	Centromere-specific multicolour FISH, subcentromere-specific M-FISH	AF	*de novo*	female/ prenatal	1. Abnormal second trimester screen (AFP3) with DS risk of 1/31;<br>2. Delivery by caesarean section for foetal distress at 40-week gestation;<br>3. Birth weight 8 lbs 1 oz length 20 1/2 inches;<br>4. Normal newborn, normal at 1 year of age;<br>5. At 1 year, ready to walk (currently walking holding on to furniture), drinks from a cup, has a neat pincer, say a mama, dada;<br>6. Weight is 20 lbs 8 oz and she is 31 inches long	Case 10-O-p12.1/1-1 [39];<br>Case 1 [50];<br>Case 60 [51]
2	47,XX,+mar[16]/ 46,XX[8]<br><br>min(10)(:p11.23 →q11.1~11.21:)<br><br>mos47,XX,+min (10)(:p11.23→ q11.1~11.21:)[16] /46,XX[8]	SKY, BACs; oligo aCGH, FISH with telomere probe	AF	*de novo*	female/ prenatal	1. IVF;<br>2. Amniocentesis at 17th week of gestation;<br>3. High-resolution ultrasound did not show any abnormality in the female foetus;<br>4. Born at 38th week of gestation;<br>5. Birth weight - 3222 g (50th centile) and height - 47.5 cm (25th centile)	Case 10-O-p11.23~ 11.22/1-1 [39,52]
3	47,XX,+mar<br><br>r(10)(::p11.21~ 11.22→q11.21~ q11.22::)	Centromere-specific multicolour FISH, subcentromere-specific M-FISH,	AF	*de novo*	female/ prenatal	1. Advanced maternal age;<br>2. Ultrasound normal;<br>3. Normal child born	Case 10-O-p11.21~ 11.22/1-1 [39];<br>Case 2 [50]

	47,XX,+r(10)(::p11.21~11.22→q11.21~q11.22::)	UPD-test					
4	47,XX,+mar[80]/46,XX[20] min(10)(:p11.21→q11.22:)<br><br>mos47,XX,+min(10)(:p11.21→q11.22:)[80]/46,XX[20]	Centromere-specific multicolour FISH, subcentromere-specific M-FISH	AF	*de novo*	female/prenatal	1. Advanced maternal age;<br>2. Ultrasound normal;<br>3. Normal child born in 33-week by section;<br>4. Birth weight 2100 g	Case 10-O-p11.21/1-1 [39];<br>Case 3 [50]
5	47,XY,+mar[15]/46,XY[35] r(10)(::p11.21→q11.21::)<br><br>mos47,XY,+r(10)(::p11.21→q11.21::)[15]/46,XY[35]	centromeric probes, subcentromere-specific M-FISH, aCGH	AF	*de novo*	male/prenatal	1. Normal child born;<br>2. At age of 10 y.o. normal	Case 10-O-p11.21/2-1 [39];<br>Case 4 [50]
6	47,XX,+mar[25]/46,XX[10]<br><br>min(10)(:p11.21→q11.21:)<br><br>mos47,XX,+min(10)(:p11.21→q11.21:)[25]/46,XX[10]	Centromere-specific multicolour FISH, subcentromere-specific M-FISH	AF	*de novo*	female/prenatal	Healthy girl was born	Case 10-O-p11.21/3-1 [39];<br>Case 2 [53]
7	47,XX,+mar/	Centromere-	AF	*de novo*	female/	1. Normal in ultrasound;	Case

	46,XX<br><br>min(10)(:p11.1→ q11.21~q11.22:)<br><br>47,XX,+min(10) (:p11.1→q11.21~ q11.22:)/ 46,XX (number of analysed cells is not clear indicated)	specific multicolour FISH, subcentromere-specific M-FISH			prenatal	2. Twin pregnancy;<br>3. Karyotype of brother normal;<br>4. Birth weight of female with marker 2280 g and of male 2350 g;<br>5. Both at 8 weeks normal	10-O-p11.1/1-1 [39];<br>Case 5 [50]
8	47,XX,+mar[100]<br><br>min(10)(:p11.21 →q11.1:)<br><br>47,XX,+min(10) (:p11.21→q11.1:)	aCGH	AF	n.a.	female/ prenatal	1. An increased risk at first-trimester screening;<br>2. The risk for trisomy 21 was 1:30;<br>3. Ultrasound normal at 20 weeks;<br>4. Normal child born in week 38;<br>5. Birth weight 3222 g, a height of 47.5 cm;<br>6. No phenotypic abnormalities at 3-years follow-up;<br>6. Started walking at 15 months and talking at 2.5 years	Case 10-O-p11.21/4-2 [39,54]
9	47,XX,+mar[49]/ 46,XX[51]<br><br>mar(10)(:p11.21 →q11.1:)<br><br>mos47,XX,+mar (10)(:p11.21→ q11.1:)[49]/ 46,XX[51]	aCGH, FISH	PBL	mat	female/ adult	1. A 40 y.o. woman;<br>2. Phenotypically normal;<br>3. No developmental delay, learning problems, or intellectual disability;<br>4. Long-term infertility;<br>5. Laboratory data showed no biological, hormonal, coagulation, or semen anomalies;<br>6. Mother (47,XX,+mar[61/46,XX[39]) and sister (47,XX,+mar[77/46,XX[23]) with sSMC normal	Case 10-O-p11.21/4-1 [39,55]

10	47,XX,+mar<br><br>min(10)(:p11.1→q11.1:) or min(10)(:p11.1→q11.1::p11.1→q11.1:)<br><br>47,XX,+min(10)(:p11.1→q11.1:)	Centromere-specific multicolour FISH, subcentromere-specific M-FISH, UPD-test	AF	*de novo*	female/ prenatal	1. Advanced maternal age;<br>2. Twin pregnancy,<br>3. Karyotype of brother normal;<br>4. Normal babies born;<br>5. Female with mar normal at 2 y.o.	Case 10-O-p11.1/2-1 [39];<br>Case 6 [50]

References from Table S3

21. Liu, R.; Hu, W.; Li, X.; Pu, D.; Yang, G.; Liu, H.; Tan, M.; Zhu, D. Association of circulating BMP9 with coronary heart disease and hypertension in Chinese populations. *BMC Cardiovasc. Disord.* **2019**, *19*, 131; DOI:10.1186/s12872-019-1095-2.
22. Mahato, A.K.; Sidorova, Y.A. RET receptor tyrosine kinase: Role in neurodegeneration, obesity, and cancer. *Int. J. Mol. Sci.* **2020**, *21*, 7108; DOI:10.3390/ijms21197108.
23. Janssens, R.; Struyf, S.; Proost, P. Pathological roles of the homeostatic chemokine CXCL12. *Cytokine Growth Factor Rev.* **2018**, *44*, 51–68; DOI:10.1016/j.cytogfr.2018.10.004.
24. Hardy, D.; Fefeu, M.; Besnard, A.; Briand, D.; Gasse, P.; Arenzana-Seisdedos, F.; Rocheteau, P.; Chrétien, F. Defective angiogenesis in CXCL12 mutant mice impairs skeletal muscle regeneration. *Skelet. Muscle* **2019**, *9*, 25; DOI:10.1186/s13395-019-0210-5.
25. Döring, Y.; van der Vorst, E.P.C.; Duchene, J.; Jansen, Y.; Gencer, S.; Bidzhekov, K.; Atzler, D.; Santovito, D.; Rader, D.J.; Saleheen, D.; et al. CXCL12 derived from endothelial cells promotes atherosclerosis to drive coronary artery disease. *Circulation* **2019**, *139*, 1338–1340; DOI:10.1161/CIRCULATIONAHA.118.037953.
26. Kim, H.; Sung, J.; Kim, H.; Ryu, H.; Cho Park, H.; Oh, Y.K.; Lee, H.-S.; Oh, K.-H.; Ahn, C. Expression and secretion of CXCL12 are enhanced in autosomal dominant polycystic kidney disease. *BMB Rep.* **2019**, *52*, 463–468; DOI:10.5483/BMBRep.2019.52.7.112.
27. De Caterina, R.; Zampolli, A. From asthma to atherosclerosis — 5-lipoxygenase, leukotrienes, and inflammation. *N. Engl. J. Med.* **2004**, *350*, 4–7; DOI:10.1056/nejmp038190.
28. Chu, J.; Praticò, D. 5-lipoxygenase as an endogenous modulator of amyloid β formation in vivo. *Ann. Neurol.* **2011**, *69*, 34–46; DOI:10.1002/ana.22234.
29. Chen, Y.; Hu, Y.; Zhang, H.; Peng, C.; Li, S. Loss of the Alox5 gene impairs leukemia stem cells and prevents chronic myeloid leukemia. *Nat. Genet.* **2009**, *41*, 783–792; DOI:10.1038/ng.389.
30. Arno, G.; Hull, S.; Robson, A.G.; Holder, G.E.; Cheetham, M.E.; Webster, A.R.; Plagnol, V.; Moore, A.T. Lack of interphotoreceptor retinoid binding protein caused by homozygous mutation ofRBP3Is associated with high myopia and retinal dystrophy. *Investig. Opthalmology Vis. Sci.* **2015**, *56*, 2358; DOI:10.1167/iovs.15-16520.
31. Rusciano, D.; Bagnoli, P. RBP3: A possible prognostic marker and therapeutic target in diabetic retinopathy. *Ann. Transl. Med.* **2019**, *7*, S327; DOI:10.21037/atm.2019.09.133.
32. Chen, C.; Grzegorzewski, K.J.; Barash, S.; Zhao, Q.; Schneider, H.; Wang, Q.; Singh, M.; Pukac, L.; Bell, A.C.; Duan, R.; et al. An integrated functional genomics screening program reveals a role for BMP-9 in glucose homeostasis. *Nat. Biotechnol.* **2003**, *21*, 294–301; DOI:10.1038/nbt795.
33. Cheng, H.; Jiang, W.E.I.; Phillips, F.M.; Haydon, R.C.; Peng, Y.; Zhou, L.A.N.; Luu, H.H.; An, N.; Breyer, B.; Vanichakarn, P.; et al. OSTEOGENIC activity of the fourteen types of human bone morphogenetic proteins (BMPS). *J. Bone Jt. Surg. Am. Vol.* **2003**, *85*, 1544–1552; DOI:10.2106/00004623-200308000-00017.
34. Song, D.; Zhang, F.; Reid, R.R.; Ye, J.; Wei, Q.; Liao, J.; Zou, Y.; Fan, J.; Ma, C.; Hu, X.; et al. BMP9 induces osteogenesis and adipogenesis in the immortalized human cranial suture progenitors from the patent sutures of craniosynostosis patients. *J. Cell. Mol. Med.* **2017**, *21*, 2782–2795; DOI:10.1111/jcmm.13193.
35. Lopez-Coviella, I. Induction and maintenance of the neuronal cholinergic phenotype in the central nervous system by BMP-9. *Science* **2000**, *289*, 313–316; DOI:10.1126/science.289.5477.313.
36. Li, S.; Dai, H.; He, Y.; Peng, S.; Zhu, T.; Wu, Y.; Li, C.; Wang, K. BMP9 inhibits the growth of breast cancer cells by downregulation of the PI3K/Akt signaling pathway. *Oncol. Rep.* **2018**, 10.3892/or.2018.6572; DOI:10.3892/or.2018.6572.
37. Song, B.; Li, X.F.; Yao, Y.; Xu, Q.Q.; Meng, X.M.; Huang, C.; Li, J. BMP9 inhibits the proliferation and migration of fibroblast-like synoviocytes in rheumatoid arthritis via the PI3K/AKT signaling pathway. *Int. Immunopharmacol.* **2019**, *74*, 105685; DOI:10.1016/j.intimp.2019.105685.
38. Tomazic, P.V.; Birner-Gruenberger, R.; Leitner, A.; Spoerk, S.; Lang-Loidolt, D. Seasonal proteome changes of nasal mucus reflect perennial inflammatory response and reduced defense mechanisms and plasticity in allergic rhinitis. *J. Proteom.* **2016**, *133*, 153–160; DOI:10.1016/j.jprot.2015.12.021.

References from Tables S5-S7

39. Liehr, T. Small supernumerary marker chromosomes. Availabe online: http://cs-tl.de/DB/CA/sSMC/0-Start.html (accessed on 8 May 2021).
40. Chen, Z.; Meloni-Ehrig, A.; Palumbos, J.C.; Guan, X.-Y.; Carroll, K.L.; Dent, K.M.; Carey, J.C. Pure trisomy 10p resulting from an extra ring chromosome: Characterization by methods of advanced molecular cytogenetics. *Am. J. Med. Genet.* **2001**, *102*, 379–382; DOI:10.1002/ajmg.1470.
41. Schlegel, M.; Baumer, A.; Riegel, M.; Wiedemann, U.; Schinzel, A. Maternal uniparental isodisomy 10 and mosaicism for an additional marker chromosome derived from the paternal chromosome 10 in a fetus. *Prenat. Diagn.* **2002**, *22*, 418–421; DOI:10.1002/pd.337.
42. Blennow, E.; Tillberg, E. Small extra ring chromosome derived from chromosome 10p: Clinical report and characterisation by FISH. *J. Med. Genet.* **1996**, *33*, 399–402; DOI:10.1136/jmg.33.5.399.

43. Trimborn, M.; Grueters, A.; Neitzel, H.; Tönnies, H. First small supernumerary ring chromosome carrying 10q euchromatin in a patient with mild phenotype characterized by molecular cytogenetic techniques and review of the literature. *Cytogenet. Genome Res.* **2004**, *108*, 278–282; DOI:10.1159/000081524.
44. Snyder, F.F.; Lin, C.C.; Rudd, N.L.; Shearer, J.E.; Heikkila, E.M.; Hoo, J.J. A de novo case of trisomy 10p: Gene dosage studies of hexokinase, inorganic pyrophosphatase and adenosine kinase. *Hum. Genet.* **1984**, *67*, 187–189; DOI:10.1007/bf00272998.
45. Lozić, B.; Čulić, V.; Lasan, R.; Tomasović, M.; Šamija, R.K.; Zemunik, T. Complete trisomy 10p resulting from an extra stable telocentric chromosome. *Am. J. Med. Genet. A* **2012**, *158A*, 1778–1781; DOI:10.1002/ajmg.a.35384.
46. Castronovo, C.; Valtorta, E.; Crippa, M.; Tedoldi, S.; Romitti, L.; Amione, M.C.; Guerneri, S.; Rusconi, D.; Ballarati, L.; Milani, D.; et al. Design and validation of a pericentromeric BAC clone set aimed at improving diagnosis and phenotype prediction of supernumerary marker chromosomes. *Mol. Cytogenet.* **2013**, *6*, 45; DOI:10.1186/1755-8166-6-45.
47. Marle, N.; Martinet, D.; Aboura, A.; Joly-Helas, G.; Andrieux, J.; Flori, E.; Puechberty, J.; Vialard, F.; Sanlaville, D.; Fert Ferrer, S.; et al. Molecular characterization of 39 de novo sSMC : Contribution to prognosis and genetic counselling, a prospective study. *Clin. Genet.* **2013**, *85*, 233–244; DOI:10.1111/cge.12138.
48. May, L.F.; Pressley, D.; Johnson, D.; Cooper, S.; Wong, A.; Martin Lese, C.; Ledbetter, D. Development and application of a pericentromeric clone set for exploring genomic instability and architecture. In Abstract book of the 54th Annual Meeting of the American Society of Human Genetics.; 2004; p. 191.
49. Sheth, F.; Andrieux, J.; Ewers, E.; Kosyakova, N.; Weise, A.; Sheth, H.; Romana, S.-P.; LeLorc'h, M.; Delobel, B.; Theisen, O.; et al. Characterization of sSMC by FISH and molecular techniques. *Eur. J. Med. Genet.* **2011**, *54*, 247–255; DOI:10.1016/j.ejmg.2011.01.011.
50. Liehr, T.; Stumm, M.; Wegner, R.D.; Bhatt, S.; Hickmann, P.; Patsalis, P.C.; Meins, M.; Morlot, S.; Klaschka, V.; Ewers, E.; et al. 10p11.2 to 10q11.2 is a yet unreported region leading to unbalanced chromosomal abnormalities without phenotypic consequences. *Cytogenet. Genome Res.* **2009**, *124*, 102–105; DOI:10.1159/000200094.
51. Huang, B.; Solomon, S.; Thangavelu, M.; Peters, K.; Bhatt, S. Supernumerary marker chromosomes detected in 100 000 prenatal diagnoses: Molecular cytogenetic studies and clinical significance. *Prenat. Diagn.* **2006**, *26*, 1142–1150; DOI:10.1002/pd.1575.
52. Sung, P.L.; Chang, S.P.; Wen, K.C.; Chang, C.M.; Yang, M.J.; Chen, L.C.; Chao, K.C.; Huang, C.Y.F.; Li, Y.C.; Lin, C.C. Small supernumerary marker chromosome originating from chromosome 10 associated with an apparently normal phenotype. *Am. J. Med. Genet. A* **2009**, *149A*, 2768–2774; DOI:10.1002/ajmg.a.32878.
53. Ou, J.; Wang, W.; Liehr, T.; Klein, E.; Hamid, A.B.; Wang, F.; Duan, C.; Li, H. Characterization of three small supernumerary marker chromosomes (sSMC) in humans. *J. Matern. Fetal Neonatal Med.* **2013**, *26*, 106–108; DOI:10.3109/14767058.2012.732129.
54. Barranco, L.; Costa, M.; Lloveras, E.; Ordóñez, E.; Maiz, N.; Hernando, C.; Villa, O.; Cirigliano, V.; Plaja, A. Three-year follow-up of a prenatally ascertained apparently non-mosaic sSMC(10): Delineation of a non-critical region. *Cytogenet. Genome Res.* **2015**, *147*, 209–211; DOI:10.1159/000444600.
55. Santacroce, R.; Trunzo, R.; Leccese, A.; Pansini, A.; Gentile, M.; Margaglione, M. The first case of a small supernumerary marker chromosome derived from chromosome 10 in an adult woman with an apparently normal phenotype. *Syst. Biol. Reprod. Med.* **2015**, *61*, 398–402; DOI:10.3109/19396368.2015.1067936.
